# Molecular Mechanisms of Basil (*Ocimum basilicum* L.) Polyphenol Extracts as Bio-Based Cryoprotectants for *Streptococcus thermophilus*: Chemical Profiling, DFT, Molecular Dynamics and Cell Viability

**DOI:** 10.3390/molecules31152661

**Published:** 2026-07-30

**Authors:** Valeria A. Pyanchenkova, Vladislav S. Filozop, Mikhail O. Volodarskiy, Dmitrii N. Borovikov, Olga L. Balabanova, Olga O. Osmak, Semen S. Kazarin, Pavel V. Nesterov, Ivan V. Moskalenko, Mariia S. Ashikhmina, Ekaterina V. Skorb

**Affiliations:** 1 Infochemistry Scientific Center, ITMO University, 9 Lomonosova Str., 191002 St. Petersburg, Russiaborovikov.d@itmo.ru (D.N.B.);; 2 Saint-Petersburg Institute of Emergency Care Named After I.I. Dzhanelidze, 192242 St. Petersburg, Russia

**Keywords:** cryopreservation, bio-based cryoprotectants, basil extract, *Ocimum basilicum*, lactic acid bacteria, *Streptococcus thermophilus*, phenolic compounds, antioxidant activity, membrane integrity, density functional theory

## Abstract

Natural plant extracts rich in polyphenols are increasingly being studied as multifunctional food ingredients with antioxidant and stabilizing properties. In this study, *Ocimum basilicum* L. extracts were evaluated as biological cryoprotective agents for *Streptococcus thermophilus*. The extracts contained high levels of phenolic compounds (~1350–2200 mg GAE equivalents/L) and exhibited strong antioxidant activity (up to 5.6 mM Trolox equivalents). Density functional theory calculations showed low O–H bond dissociation energies (~72–74 kcal/mol in ethanol) for key components, including luteolin and rosmarinic acid. These calculations indicate a high hydrogen donation capacity comparable to or exceeding that of ascorbic acid. Molecular dynamics simulations demonstrated the preferential localization of major phenolic compounds at the membrane–water interface in a POPC bilayer membrane. The interaction of molecules with POPC increased membrane thickness and formed stable hydrogen-bond networks with lipid head groups. Experiments showed that systems based on basil extract significantly increased the survival of bacteria after storage at −25 °C, with the number of viable cells reaching (1.5–2.75) × 10^8^ CFU/mL. This effect was observed in comparison with control groups that used saline or sucrose. Fluorescent analysis of live/dead cells confirmed the improvement in cell membrane preservation. At the same time, no signs of metabolic inhibition were detected. Taken together, the experimental and computational results support the hypothesis that the cryoprotective effect may involve complementary antioxidant and membrane-associated interactions. However, direct biophysical validation of the proposed membrane mechanism is still required. These results emphasize that polyphenol extracts are promising natural functional ingredients for improving the stability and shelf life of probiotic and starter cultures in food systems.

## 1. Introduction

The development of safe, bio-based approaches for microbial preservation has become an important focus in food biotechnology. In this context, special attention is paid to methods that minimize the use of synthetic additives and ensure high biocompatibility of auxiliary components used in the storage and processing of bacterial cultures.

Cryopreservation is widely used for long-term storage of microorganisms; however, freezing and thawing induce multiple stress factors, including ice formation, dehydration, and oxidative damage. At the same time, freezing and subsequent thawing are accompanied by several stress factors, including water crystallization, dehydration, and osmotic stress. These processes can lead to intracellular ice formation and mechanical damage to cellular structures. An additional factor of damage is oxidative stress, which occurs when the oxidation process exceeds antioxidant protection and leads to the formation of reactive oxygen species (ROS). ROS disrupt membrane integrity, induce lipid peroxidation, and damage cellular macromolecules [1,2].

Cryoprotectors bind to protein functional groups through ionic and hydrogen bonding interactions. This binding reduces the availability of proteins to free water molecules, which reduces the likelihood of their denaturation and aggregation during freezing and thawing. The most widely used penetrating cryoprotectors are glycerin, dimethyl sulfoxide (DMSO), and ethylene glycol, which effectively inhibit ice crystal formation and reduce cellular damage at low temperatures [3]. However, these compounds can have toxic effects on cells and increase osmotic stress, necessitating their removal after thawing, which complicates the technological process and reduces its practical applicability [4,5].

Recent advances in the preservation of *Streptococcus thermophilus* have shifted from the use of individual protective agents toward optimized multicomponent formulations and mechanism-based cryoprotective strategies. A combination of 6% sucrose, 8% skim milk, and 4% sodium glutamate provided a freeze-drying survival rate of 90.59% for *S. thermophilus* 937, demonstrating the synergistic action of protectants with different functions [6]. Antifreeze peptides have also been proposed as promising cryoprotective agents. A recombinant snow flea antifreeze peptide was shown to preserve membrane permeability, fluidity, structural integrity, and membrane potential by interacting with the bacterial cell surface and modifying extracellular ice formation during freezing [7]. More recently, an optimized direct-vat-set formulation containing skim milk, sodium glutamate, and trehalose improved the post-freeze-drying viability and fermentation performance of *S. thermophilus* Q-1 [8]. These findings indicate that effective preservation requires the simultaneous control of ice formation, dehydration, and membrane damage. However, the potential of plant-derived polyphenol-rich extracts as multifunctional cryoprotectants for *S. thermophilus* remains insufficiently explored.

In recent years, there has been increasing interest in natural, bio-based cryoprotectors with antioxidant and membrane-stabilizing properties [2]. Natural phenolic compounds, including phenolic acids, flavonoids, essential oils, and terpenes, are considered promising components of cryoprotective systems, although most studies have focused on eukaryotic cells [9,10,11]. Attention has been given to natural deep eutectic solvents (NADESs), which exhibit high biocompatibility and cryoprotective activity due to their ability to inhibit ice formation and modify the structural properties of water [2,9]. NADESs have also been shown to enhance the antioxidant activity of plant metabolites by forming stable hydrogen-bond networks and stabilizing biomolecules under stress conditions [2,12].

Despite significant progress in developing bio-based cryoprotectors, data on their effectiveness against bacteria remains limited. This gap is significant for the food and biotechnology industries, where safe and practical approaches to conserving probiotic and technologically significant microorganisms are needed.

Plant extracts containing polyphenols, flavonoids, phenolic acids, and terpene compounds exhibit pronounced antioxidant and membrane-stabilizing properties, making them promising candidates for protecting bacterial cells during cryopreservation [13].

## 2. Results

### 2.1. Chlorophyll Extraction and Removal

**Figure 1:**
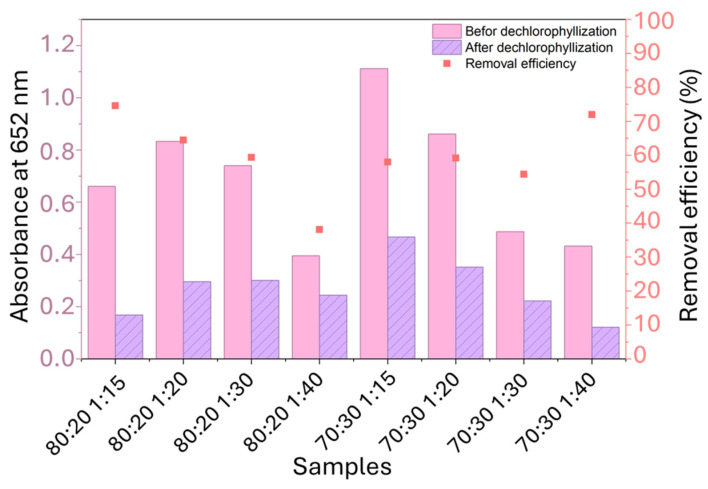
Efficiency of chlorophyll removal from basil extracts (λ = 652 nm). Changes in the optical density of basil extracts before and after the dechlorophyllation stage and the corresponding chlorophyll removal efficiency for various extraction conditions.

Basil leaves were extracted with aqueous ethanol solutions (80%, 70%) at different solvent ratios (1:15–1:40), and intensely colored extracts with high optical density values at 652 nm were obtained (Figure 1).

The use of low-temperature freezing followed by cold filtration resulted in a 2- to 4-fold decrease in A652 compared with the initial extracts. The calculation of chlorophyll removal efficiency according to Formula (1) showed that, across all considered extraction samples, this indicator exceeded 60%. For extracts with a 70:30 ratio of 1:30–1:40, the maximum values were observed. The combination of water–ethanol extraction and subsequent low-temperature dechlorophyllation ensures a high degree of chlorophyll removal while maintaining the dissolved phenolic fraction.

### 2.2. Antioxidant Activity and Total Content of Phenolic Compounds

**Figure 2:**
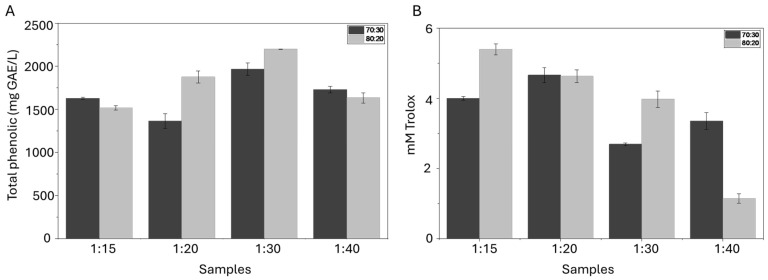
(**A**) is the total content of phenolic compounds in the samples determined by the Folin–Chocalteu method and expressed in mg of gallic acid equivalent per liter (mg GAE/L). (**B**) is the antioxidant activity of basil extracts according to the DPPH test, expressed in Trolox equivalents (mM), at different ratios (1:15–1:40) and ethanol concentrations. The values are given as the mean ± standard deviation (*n* = 3).

The antioxidant activity of the extracts, expressed in Trolox equivalents, varied depending on the ethanol concentration in the extraction mixture and its ratio (Figure 2A). Increased inhibition of the DPPH radical was observed for all samples compared with the control, which confirms the presence of biologically active compounds in the extracts capable of neutralizing free radicals, slowing down oxidative processes.

The highest antioxidant activity was observed for the extract obtained with 80% ethanol at a 1:15 ratio (5.6 mM Trolox). Upon transition to 1:20, the activity of the 70% and 80% extracts becomes comparable (4.5–4.7 mM Trolox), indicating partial saturation of the extracts with antioxidant metabolites. At 1:30, a higher activity level was observed for the 80% sample (~4.0 mM Trolox versus ~2.7 mM for 70%), whereas at 1:40, higher values were observed for 70% (~3.4 mM) compared with 80% (~1.1 mM). This nonlinear relationship indicates the joint contribution of solvent polarity and the degree of raw material dilution to the extraction efficiency of individual subgroups of phenolic and related compounds.

The total content of phenolic compounds, determined by the Folin–Chocalteu method and expressed in mg GAE/L, ranged from 1350 to 2200 mg GAE/L (Figure 2B). The minimum phenolic content was observed for 70% of the extract at 1:20 (~1350 mg GAE/L), and the maximum for 80% at 1:30 (~2200 mg GAE/L). For 1:15, the phenol content was ~1650 mg GAE/L (70%) and ~1500 mg GAE/L (80%), whereas for 1:40 (70% and 80%), it was 1750 and 1650 mg GAE/L, respectively.

A comparison of data on antioxidant activity and total phenolic content showed that a proportional increase in radical-scavenging ability did not always accompany a high phenolic concentration. In some cases (for example, at 70% and 80% at 1:30), a significant content of phenolic compounds was observed, along with moderate antioxidant activity. Thus, the total antioxidant effect is mainly determined by individual structural subgroups of polyphenols (flavones, phenolic acids, phenylpropanoids), which differ in their abilities to operate via HAT and SET mechanisms, as well as by the possible presence of concomitant components with lower antioxidant activity.

### 2.3. Identification of Compounds in the Extract by HPLC-ESI-MS/MS

**Figure 3:**
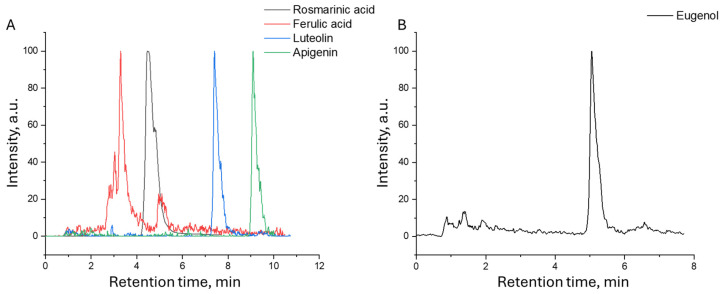
Representative MRM chromatograms of phenolic compounds identified in basil (*Ocimum basilicum* L.) extract by HPLC–ESI–MS/MS: (**A**) rosmarinic acid (gray), ferulic acid (red), luteolin (blue), and apigenin (green); (**B**) eugenol (black). Phenolic acids and flavonoids were detected in negative-ionization mode (ESI−), whereas eugenol was analyzed in positive-ionization mode (ESI+). Identification was based on retention times and MRM transitions using reference standards.

A qualitative analysis of basil extracts (*Ocimum basilicum* L.) performed by HPLC-ESI-MS/MS confirmed the presence of several key phenolic compounds, including phenolic acids and flavonoids (Figure 3). The compounds were identified based on the coincidence of retention time and characteristic MRM transitions with standard samples.

Chromatograms for rosmarinic acid, ferulic acid, luteolin, and apigenin were obtained in the negative-ionization regime (ESI−). Among the identified substances, rosmarinic acid showed the highest signal intensity. At the same time, luteolin and apigenin were recorded at higher retention times. The achieved chromatographic separation ensured reliable qualitative identification of the studied compounds. In the positive-ionization mode (ESI+), a characteristic peak for eugenol was observed.

The data obtained indicate that basil extracts contain a complex of phenolic compounds of various structural classes. The predominance of rosmarinic acid and the presence of flavonoids such as luteolin and apigenin are consistent with the literature data on the phytochemical composition of basil.

The identified compounds have pronounced antioxidant activity, which may contribute to the observed cryoprotective effect of the studied extracts. These compounds were selected as model compounds for subsequent quantum chemical analysis (DFT) to assess their antioxidant potential at the molecular level.

### 2.4. DFT Calculations of the Antioxidant Potential of Phenolic Compounds

**Figure 4:**
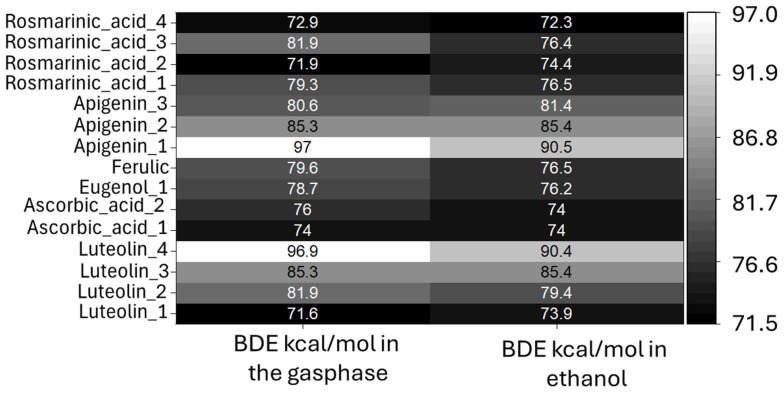
Heat map of the values of the O–H bond-breaking energy (BDE, calculated using Gibbs energy) for individual phenolic radicals in the gas phase and in ethanol, reflecting the influence of the medium on the thermodynamic preference of hydrogen atom donation.

For the main phenolic components of the extracts (luteolin, apigenin, rosmarinic and ferulic acids, eugenol), the O–H bond-breaking energies (BDE, Gibbs energy) in the gas phase and in ethanol, gaps between boundary orbitals (HOMO–LUMO) and maps of the molecular electrostatic potential (MEP) were calculated (Figure 4, Table 1).

According to quantum chemical modeling data (Table 2), the studied phenolic compounds in basil extracts differ significantly in thermodynamic parameters that determine their antioxidant properties.

The minimum values of BDE (Gibbs energy) in the gas phase were obtained for luteolin (OH_1_ radical, ~72 kcal/mol) and rosmarinic acid (OH_2_ and OH_4_ radicals, ~72 kcal/mol). For the same centers in ethanol, BDE increases slightly (up to ~74 kcal/mol), while maintaining their ranking as the most thermodynamically advantageous hydrogen atom donors. In ascorbic acid, the BDE of OH groups occupies an intermediate position (≈74–76 kcal/mol), whereas for ferulic acid and eugenol, the BDE values are higher (~79–80 kcal/mol), and for some phenolic centers of apigenin, they reach ~97 kcal/mol. Thus, the least strong O–H bonds are localized in luteolin and rosmarinic acid, and the most durable in apigenin.

The low BDE values for individual hydroxyl groups of luteolin and rosmarinic acid are explained by the developed pi conjugation system and the possibility of effective resonance stabilization of the formed radical. In the case of luteolin, the most active center is located in the B-ring, which contains the ortho-diphenolic fragment. For apigenin, which lacks one hydroxyl group in the B-ring, the minimum BDE values shift to other phenolic centers (OH_2_/OH_3_, ~80–85 kcal/mol), and the overall ability to neutralize radicals by the HAT mechanism is lower than that of luteolin. For rosmarinic acid, the presence of two aromatic fragments and several phenolic groups leads to multiple hydrogen-donating centers, and O–H bonds (OH_2_ and OH_4_) can provide multi-stage radical protection.

A BDE value below ≈80 kcal/mol indicates a property characteristic of highly active hydrogen atom donors; i.e., luteolin and rosemary are comparable or superior to ascorbic acid. The phenolic centers of eugenol and ferulic acid are less prone to breaking the O–H bond, potentially leading to more moderate antioxidant activity via the HAT mechanism and explaining their secondary effect on the extract’s antioxidant activity.

The inclusion of the solvent effect (CPCM model for ethanol) led to a change in the absolute BDE values of 2–4 kcal/mol. However, it did not alter the qualitative order of reactivity among various centers and compounds. In ethanol, the key hydroxyl groups of luteolin and rosmarinic acid showed the lowest enthalpy and free-energy values for the BDE. Thus, the solvent effect is predominantly stabilizing and does not alter the set of key donor O–H groups.

**Figure 5:**
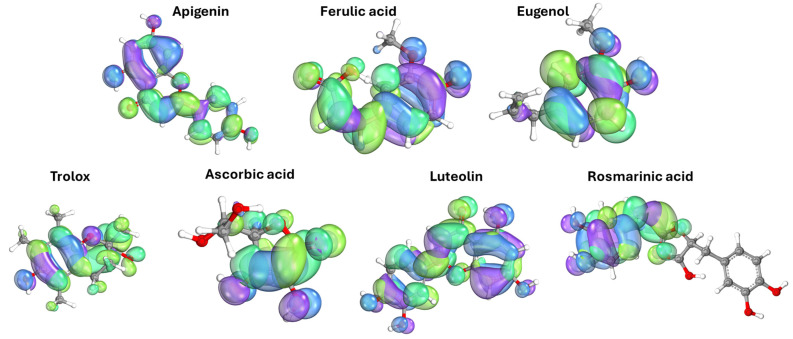
Electron density distribution surfaces of the highest occupied molecular orbital (HOMO) and the lowest unoccupied molecular orbital (LUMO) for apigenin, ferulic acid, eugenol, luteolin, rosmarinic acid, ascorbic acid, and trolox, calculated at the B3LYP-D4/CPCM level. HOMO is shown in green, whereas LUMO is shown in purple. The orbital distributions illustrate the localization of electron-rich and electron-accepting regions in the investigated molecules.

Analysis of the energy characteristics of the boundary orbitals further confirmed the high electron-donating ability of the selected compounds. The energy gaps between the highest occupied and lowest unoccupied molecular orbital (HOMO-LUMO gap) for luteolin, apigenin, ferulic acid, and rosmarinic acid in the gas phase were in the range of 0.151–0.18 Eh (Table 2, Figure 5), while for ascorbic acid, this parameter was higher (≈0.20 Eh), indicating an increased ability for single-electron transfer (SET mechanism). Eugenol showed a decrease in the HOMO-LUMO gap to ~0.10 Eh, indicating an increased ability to utilize the SET mechanism in a polar environment. The combination of a low HOMO-LUMO gap and low BDEs in luteolin and rosmarinic acid indicates their ability to implement both HAT and SET mechanisms efficiently.

**Figure 6:**
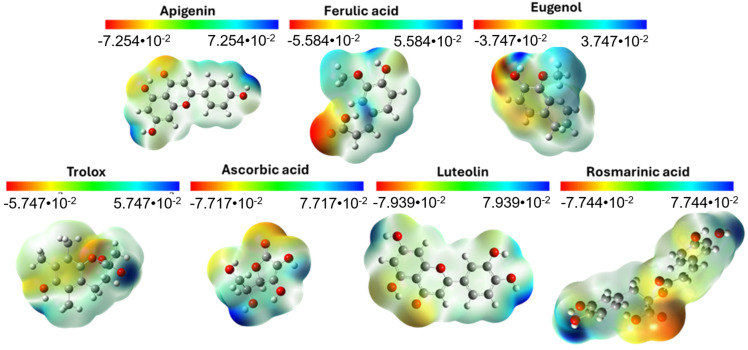
Molecular electrostatic potential (MEP) maps ofthe investigated phenolic compounds. Regions of negative electrostatic potential (red) indicate electron-rich sites, whereas regions of positive electrostatic potential (blue)indicate electron-deficient sites potentially involved inintermolecular interactions.

The maps of the molecular electrostatic potential (MEP) (Figure 6) show that the regions of maximum negative potential in luteolin and rosmarinic acid molecules are concentrated near phenolic oxygens and conjugated aromatic fragments, forming nucleophilic regions. For eugenol and ferulic acid, the electrophilic centers are localized around a single phenolic fragment, limiting the number of potential reaction centers; thus, the MEPs are consistent with the BDE data and indicate a set of active sites in the molecules.

### 2.5. Effect of Basil Extract on the Survival of Streptococcus thermophilus During Freezing

The combination of calculated bond dissociation energy (BDE), chemical reactivity, and electrostatic potential distributions showed that luteolin and rosmarinic acid have the highest antioxidant activity due to minimal BDE values of individual O–H bonds, low HOMO-LUMO gaps, pronounced delocalization of spin density, and the presence of areas of negative electrostatic potential. Ascorbic acid occupies an intermediate position in terms of antioxidant characteristics, whereas apigenin, eugenol, and ferulic acid are characterized by a lower ability to neutralize free radicals.

Given the multifactorial nature of cell damage during cryopreservation and the previously shown role of oxidative processes in reducing the viability of microorganisms, it was of interest to evaluate the possible contribution of antioxidant components of basil extract to the preservation of bacterial cells during freezing and subsequent thawing. Lactic acid bacteria *Streptococcus thermophilus* were used as the test culture, and the culture’s resistance to cryogenic stress was evaluated in various cryoprotective media: saline solution (negative control), sucrose solution (positive control), basil extract, and combined systems containing basil extract and sucrose.

**Figure 7:**
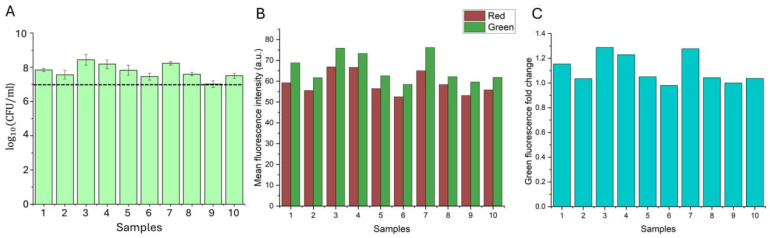
(**A**) Viability of *Streptococcus thermophilus* after storage at −25 °C for 7 days in different cryoprotective media. (**A**) Viable cell counts determined by the pour-plate method and expressed as CFU/mL. (**B**) Mean fluorescence intensity in the green and red channels after live/dead staining with LUCs 13 and YODI 3, respectively. (**C**) Green fluorescence intensity normalized to the 0.9% NaCl control, which was set to 1.0. Sample compositions are provided in Table 2. Data are presented as the mean ± standard deviation of three independent experiments (*n* = 3). Different letters indicate statistically significant differences according to one-way ANOVA followed by Duncan’s post hoc test (*p* < 0.05).

An analysis of cell survival after storage at −25 °C (Figure 7A) showed that saline and sucrose solutions provided comparable indicators of cell preservation, suggesting limited cryoprotective effectiveness of sucrose under the experimental conditions (Table 2 shows the sample numbers and experimental conditions). These samples were used as baselines for further comparison. The variants containing basil extract generally showed a tendency to increase cell survival compared to both control samples; however, the effect’s severity significantly depended on the extract concentration, the degree of dilution, and the presence of sucrose.

The most stable and reproducible survival rates were obtained with basil extract at 70% concentration and a 1:30 dilution (Figure 7A). Variants with a higher extract concentration (80%) and a lower dilution (1:15) showed reduced effectiveness in some cases. Combined systems containing basil extract and sucrose, in some variants, demonstrated greater cell safety than using either component separately.

The results of the fluorescence analysis based on differential staining of living and dead cells are shown in Figure 7B. In the control samples of NaCl and sucrose, the average intensities of green and red fluorescence were similar, as were the G/R ratios, reflecting comparable states of the cell populations after freezing. In samples with basil extract, characterized by higher survival, an increase in green fluorescence intensity was observed, corresponding to cells with preserved membrane integrity (Figure 7B).

An analysis of the relative intensity of green fluorescence normalized to saline (green fold NaCl) showed that an increase in this indicator was observed in all systems, except the variant with an extract of 70% at a dilution of 1:30 without sucrose, the values of which were comparable to the control level (Figure 7C). The highest values of green fold NaCl were recorded for systems with basil extract at a dilution of 1:30 in the presence of sucrose, as well as for the variant with 80% sucrose-free extract (Figure 7C).

A comparison of microbiological accounting data (Figure 7A) and fluorescence analysis (Figure 7B,C) revealed a consistent trend: systems that provided higher cell survival were characterized by an increased green fluorescent signal and a higher relative proportion of cells with intact membranes. At the same time, the quantitative differences between the methods may reflect the fact that fluorescence analysis primarily reflects cellular structure and can identify subpopulations of potentially viable but uncultivated cells.

### 2.6. The Effect of Basil Extract on the Acid-Forming Activity of Streptococcus thermophilus

**Figure 8:**
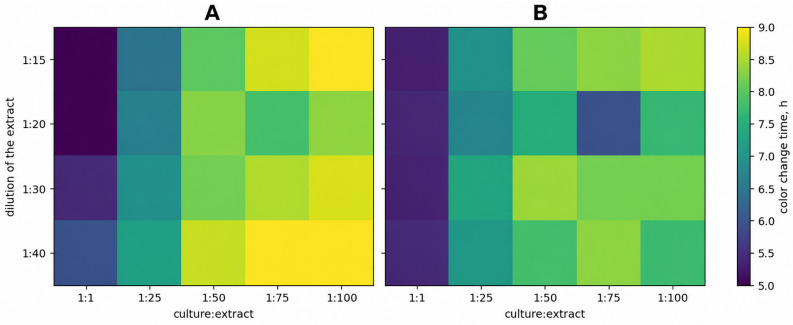
Effect of basil extract on the acid-forming activity of *Streptococcus thermophilus*, assessed by the time required for bromocresol purple to change from purple to yellow. Heat maps show the results for extracts obtained using (**A**) 70% and (**B**) 80% ethanol at solid-to-solvent ratios of 1:15, 1:20, 1:30, and 1:40 and subsequently tested at dilutions ranging from 1:1 to 1:100. The cultures were incubated in M17 medium containing bromocresol purple at 37–38.5 °C for 24 h. A sample containing an equivalent volume of 0.9% NaCl instead of basil extract was used as the control. The color scale represents the mean time to indicator color change, expressed in hours. Data were obtained from three independent experiments (*n* = 3).

Evaluation of the minimum inhibitory concentration (MIC) of basil extract by changing the color of the bromocresol purple indicator showed that the effect of the extract on the acid-forming activity of *Streptococcus thermophilus* is dose-dependent (Figure 8). In all variants with basil extract (mixtures of 70% and 80%, dilution of stock extract 1:15–1:40, ratio 1:1–1:100), the time to color change was in the range of 5–10 h and in all cases was shorter than the color change in the control medium (0.9% saline solution, color change occurred after 12 h and 15 min). Thus, in the studied concentration range, basil extract had no significant effect on the growth and metabolism of *S. thermophilus*; the minimum inhibitory concentration was not achieved.

### 2.7. MD Interactions of Extract Components with the POPC Membrane

MD modeling was performed to gain a deeper understanding of the molecular mechanisms underlying the membrane-stabilizing and cryoprotective effects of the biologically active components of basil extract. We studied the effect of a number of compounds identified or related to the key components of the extract (apigenin, eugenol, ferulic acid, luteolin, rosmarinic acid), as well as substances used as reference antioxidant molecules in DFT calculations (ascorbic acid and trolox) and classical cryoprotectants (glycerol, sucrose) for cryopreservation analysis, on the structural and dynamic properties of a model lipid membrane made of phosphatidylcholine (POPC).

**Figure 9:**
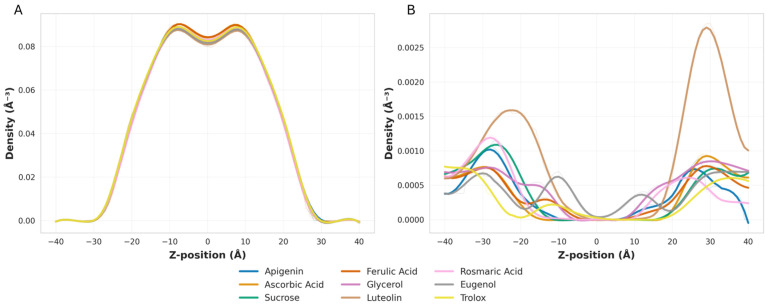
Time-averaged density of membrane molecules (POPC) (**A**) and molecules of the substances under study (**B**) in the simulation cell.

Analysis of spatial density profiles averaged over three replicas of the production simulation revealed the nature of the localization of the molecules under study relative to the lipid bilayer (Figure 9). None of the compounds showed appreciable density at the bilayer midplane (z ≈ 0), indicating that the hydrophobic core remained essentially inaccessible to all of them on the simulated timescale (Figure 9B). The compounds nevertheless differed markedly in how closely they approached the bilayer. Eugenol was the only compound to show appreciable density within the bilayer interior (|z| ≈ 10 Å), which is consistent with it being the least polar molecule of the series. The remaining compounds were localized predominantly at the water–lipid interface (|z| ≈ 25–30 Å) and in the adjacent aqueous phase. Luteolin displayed by far the highest interfacial density, exceeding that of the other compounds approximately threefold at its maximum. This is particularly notable given that the luteolin system contained the smallest number of solute atoms among the flavonoids studied, indicating a genuinely pronounced accumulation at the membrane surface rather than an effect of solute loading.

**Figure 10:**
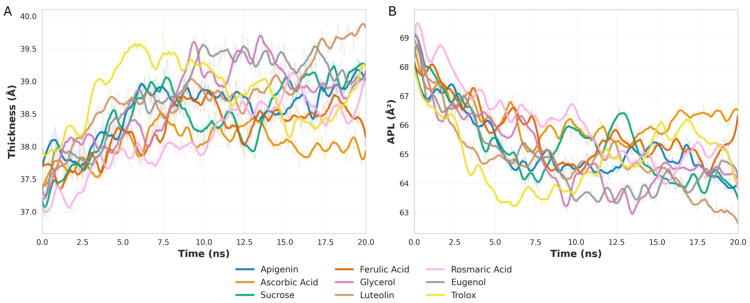
Membrane thickness (**A**) and area per lipid (APL) (**B**) during simulation for the substances under investigation.

Quantitative assessment of the effect of compounds on the macroscopic parameters of the bilayer showed significant modulation of its structural characteristics (Figure 10). The greatest changes in membrane thickness and area per lipid molecule (APL) were recorded in the presence of luteolin, rosmarinic acid, ferulic acid, and Trolox. In particular, a statistically significant increase in APL was observed for systems with these compounds, indicating a disruption of the dense packing of lipid acyl chains and the incorporation of the compound molecule into the lipid matrix. At the same time, an increase in bilayer thickness was observed for some systems, which can be interpreted as the ordering of acyl chains and a decrease in their fluctuational mobility, or as a consequence of intermonolayer expansion due to the localization of the compound in the head-group region. These changes are consistent with a potential membrane-modulating effect; however, their contribution to membrane stabilization under freezing conditions requires direct experimental validation.

The systems were matched by mass fraction rather than by molar concentration (Table S1), and this was taken into account when comparing compounds directly. Notably, the two structurally related flavones were present at comparable amounts, with luteolin at a slightly lower molar concentration than apigenin (33.3 vs. 38.9 mM); the stronger bilayer perturbation observed for luteolin therefore cannot be attributed to a higher solute loading and is instead consistent with the effect of its additional hydroxyl group. Similarly, rosmarinic acid produced pronounced changes in APL and bilayer thickness at the lowest molar concentration of all compounds studied (27.7 mM), reinforcing the conclusion that its membrane activity is intrinsically high rather than a consequence of its abundance in the system.

**Figure 11:**
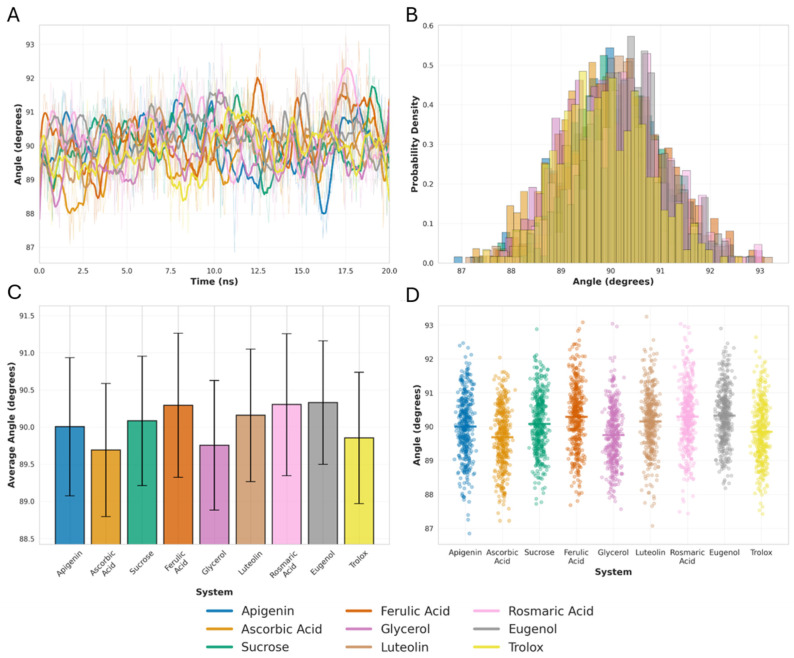
Distribution of lipid head groups depending on the molecules of the studied substances. (**A**) Distribution over the simulation time. (**B**) Angle probability density. (**C**) Average angle over the entire simulation for each system (bar chart). (**D**) Average angle over the entire simulation for each system (scatter plot).

To analyze the influence of the molecules under study on the orientation of lipid head groups, the distribution of the tilt angles of the head groups in model membrane systems was examined. Figure 11 shows the dynamics of angle changes over time, their statistical distributions, and the average values for the entire trajectory of molecular dynamics modeling for each system.

**Figure 12:**
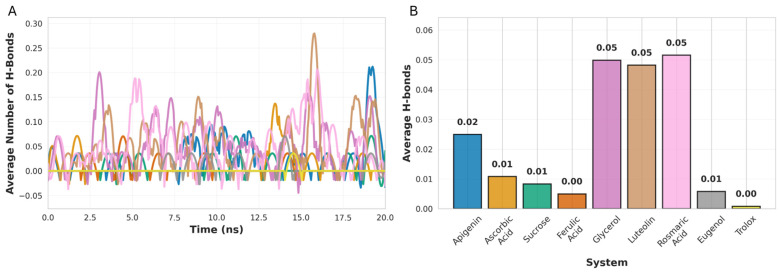
Average number of hydrogen bonds depending on the molecules studied (**A**) throughout the simulation time and (**B**) averaged over the simulation time.

Data on the dynamics of hydrogen bonds (Figure 12) directly confirmed the formation of specific molecular contacts between the extract components and the membrane. Calculation of the average number of hydrogen bonds formed by each molecule of the compound with the atoms of the lipid bilayer revealed clear differences between the compounds. Rosmarinic acid and luteolin demonstrated the greatest ability to form stable hydrogen bonds, which is fully consistent with DFT modeling data predicting minimal O–H bond dissociation energy (BDE) values and the presence of multiple active hydroxyl groups for these compounds.

The formation of a network of hydrogen bonds on the membrane surface and in the adjacent hydrophobic region can perform several protective functions: (1) stabilization of lipid head groups and reduction in their mobility, which increases the mechanical resistance of the membrane to bending deformations; (2) local replacement of water molecules in the lipid hydration shell, which can mitigate the effects of dehydration during extracellular freezing; (3) direct stabilization of membrane proteins through interaction with their surface groups.

## 3. Discussion

The results indicate that the cryoprotective effect of basil extract against *Streptococcus thermophilus* is due to the combined action of several interrelated mechanisms and is significantly influenced by the concentration of phenolic compounds and the physico-chemical state of the extract. Cryodamage is a multifactorial process involving oxidative and membrane-related stress [14,15,16].

The low survival rate of cells in saline solution confirms that the absence of components that stabilize the aqueous phase and suppress oxidative stress leads to pronounced cryoinduced damage. The use of sucrose provided moderate cell protection, consistent with its role as a non-penetrating cryoprotector that stabilizes membranes and proteins through hydrogen bonding and a partially glassy extracellular environment. However, sucrose’s effectiveness was inferior to that of systems containing basil extract, indicating the presence of additional protective mechanisms beyond purely osmotic and structural stabilization [17].

The pronounced cryoprotective effect of basil extract, especially at a ratio of 70:30 and a dilution of 1:30, may be directly related to its antioxidant properties. The extracts showed a high capacity to neutralize free radicals in the DPPH test and a significant content of phenolic compounds, indicating their potential to suppress ROS generated during freeze–thaw cycles. Oxidative stress is considered one of the key factors in cryoprotection, since ROS initiate lipid peroxidation, leading to disruption of membrane integrity, loss of ion homeostasis, and enzyme inactivation. A decrease in ROS levels is thus causally related to increased cell viability after freezing [16].

Previous studies on the preservation of *Streptococcus thermophilus* have mainly focused on classical multicomponent cryoprotective formulations and antifreeze peptides. Di et al. reported that a formulation containing 6% sucrose, 8% skim milk, and 4% sodium glutamate increased the freeze-drying survival rate of *S. thermophilus* 937 to 90.59%, demonstrating the advantages of combining compounds that reduce dehydration and stabilize cellular structures [6]. Antifreeze peptides have also been shown to protect *S. thermophilus* by modifying extracellular ice formation and preserving membrane permeability, fluidity, and metabolic activity during freezing [7,18]. In contrast, the basil polyphenol extract investigated in the present study is not expected to act primarily through direct regulation of ice growth. Based on the antioxidant assays and computational results, its proposed protective contribution is mainly associated with radical scavenging and interactions with the membrane–water interface. Thus, basil polyphenols may provide a mechanism complementary to that of conventional cryoprotectants. However, direct quantitative comparison between studies should be made with caution because of differences in bacterial strains, freezing protocols, storage conditions, and methods used to assess viability.

Plant polyphenols can directly interact with lipid bilayers, modulating membrane structure, including lipid packing and fluidity [19]. These interactions depend on the molecular structure of the compounds and contribute to membrane stabilization beyond their antioxidant activity [20].

Cold-induced oxidative responses have also been documented in *Ocimum basilicum* itself. Rezaie et al. showed that cold stress altered total phenolic and flavonoid contents, antioxidant enzyme activity, phenylalanine ammonia-lyase activity, and the expression of genes involved in phenylpropanoid biosynthesis [21]. More recently, Rodeo and Mitcham reported that basil genotypes with lower chilling sensitivity contained higher levels of soluble phenolic compounds and ascorbic acid, indicating an association between non-enzymatic antioxidant capacity and chilling tolerance [22]. Metabolomic analysis of basil leaves stored at 4 °C further revealed activation of antioxidant defense pathways together with a decrease in rosmarinic acid and ascorbic acid, which may reflect their consumption during reactive oxygen species scavenging [23]. Although these plant responses cannot be directly extrapolated to bacterial cryopreservation, they support the biological plausibility that basil phenolic compounds participate in protection against low-temperature oxidative damage. In the present system, therefore, the identified phenolic compounds may contribute primarily by limiting oxidative injury during freezing rather than by acting as classical modifiers of ice formation.

Biophysical studies of model membranes confirm that many phenolic acids and flavonoids are capable of altering the dynamic characteristics of the lipid bilayer, in some cases reducing its fluidity and increasing its structural stability. These effects correlate with the number of hydroxyl groups and the polarity of the molecules, which determines the depth of their inclusion and preferred localization in the membrane. Changes in phase transition temperatures and lipid orderliness, revealed by fluorescence spectroscopy and differential scanning calorimetry, indicate the potential physiological significance of such interactions [19,24].

Unlike polyphenols, sugars and polyols mainly exert a membrane-protective effect through an indirect hydration mechanism. According to experimental and molecular dynamics studies, sucrose at moderate concentrations interacts mainly with polar lipid heads, forming a hydrogen-bonded protective layer, whereas at higher concentrations, it localizes mainly in the aqueous phase without penetrating the membrane [25,26].

Sucrose and phenolic compounds protect bacterial cells through distinct but complementary mechanisms. Sucrose is a classical non-penetrating cryoprotectant whose main effect is associated with the physical state of the aqueous phase. It reduces water availability, increases the viscosity of the extracellular medium, promotes the formation of an amorphous protective matrix, and helps preserve the hydration of membrane phospholipids and proteins during freezing and dehydration. In contrast, phenolic compounds are not expected to act primarily by controlling ice formation. Their proposed protective role is mainly associated with the scavenging of reactive oxygen species, inhibition of lipid peroxidation, and interactions with polar lipid head groups at the membrane–water interface. Therefore, sucrose primarily limits ice- and dehydration-related damage, whereas basil polyphenols may reduce oxidative damage and contribute to the preservation of membrane integrity. The combination of these components may provide complementary protection against different types of cryoinjuries.

This mechanism, often described as “water replacement,” helps preserve membrane structure under conditions of dehydration and osmotic stress.

Less polar phenolic compounds, such as eugenol and ferulic acid, exhibit a different nature of interaction with membranes. Partial incorporation of eugenol into the hydrophobic tails of lipids is accompanied by local packing disturbances and increased bilayer permeability, which is consistent with experimental data obtained on model liposomes and LUVs [27,28].

Ferulic acid, on the contrary, is largely localized near the membrane surface and, depending on the lipid composition, exhibits a predominantly stabilizing and antioxidant effect without disturbing the overall integrity of the bilayer [29].

The key molecular factor determining the membrane-stabilizing effect of polyphenols is the formation of stable hydrogen bonds with the polar heads of lipids. Such interactions promote head layer condensation and reduce lipid mobility, while providing protection against peroxidation. Experimental data show that compounds such as rosmarinic acid effectively inhibit the lipid peroxidation reaction by interacting with the membrane surface without disrupting the structural integrity of the bilayer [30].

The molecular effects identified in MD modeling are consistent with DFT calculations and experimental studies of the antioxidant activity of polyphenols. Their ability to donate electrons or hydrogen atoms ensures local neutralization of reactive oxygen species near the most vulnerable areas of the membrane-unsaturated fragments of lipid tails and phospholipid heads.

Although the live/dead staining results indicated improved preservation of bacterial membrane integrity in the presence of basil extract, this method does not directly assess membrane fluidity, lipid ordering, bilayer thickness, or the interaction of individual phenolic compounds with the bacterial membrane. Moreover, the POPC bilayer used in the MD simulations represents a simplified model and does not fully reproduce the lipid composition of the *Streptococcus thermophilus* membrane. Therefore, the MD results should be interpreted as a molecular-level hypothesis supporting a possible membrane-associated protective mechanism rather than as direct experimental proof. Further studies using model membrane systems, fluorescence anisotropy, Laurdan-based membrane fluidity analysis, or other biophysical methods are required to confirm the effects of basil extract and individual phenolic compounds on membrane organization under freezing conditions.

The combination of the interface localization of polyphenols and osmoprotective hydration created by sucrose or glycerol forms a pronounced synergistic effect. As part of this effect, sugars and polyols maintain membrane hydration and reduce the potential toxicity of high local concentrations of phenolic compounds, while polyphenols reduce the risk of peroxidation and increase the structural stability of the bilayer [25,30,31].

The reduced protective effect observed for the extract obtained with 80% ethanol at a solid-to-solvent ratio of 1:15 suggests a concentration-dependent response rather than a direct positive relationship between total phenolic content and cryoprotective efficiency. At moderate concentrations, phenolic compounds may reduce oxidative injury by scavenging reactive oxygen species and limiting lipid peroxidation. At higher local concentrations, however, some extract components may disturb membrane organization or promote oxidative reactions under specific redox conditions. Eugenol, for example, has been shown to cause concentration-dependent membrane depolarization and permeabilization, as well as ROS-associated membrane damage in bacterial cells [32,33]. Rosmarinic acid may also exhibit pro-oxidant activity under specific conditions, particularly in the presence of redox-active components such as transition metal ions or NADH [34]. Therefore, the lower survival observed for the more concentrated extract may reflect a shift from protective antioxidant activity toward membrane-perturbing, cytotoxic, or potentially pro-oxidant effects. However, because ROS generation, lipid peroxidation, residual ethanol content, pH, and osmolarity were not directly measured for each cryoprotective formulation, the present results do not allow these mechanisms to be distinguished conclusively. Thus, the proposed pro-oxidant effect should be considered a hypothesis requiring further experimental validation.

The systems that combined basil extract with sucrose demonstrated higher and more stable survival than sucrose alone, in some cases exceeding that of the pure extract. Sucrose probably reduces osmotic and membrane stress, reduces the toxicity of concentrated phenolic components, and provides antioxidant protection. This functional complementarity corresponds to modern concepts of combined cryoprotective systems, in which different components act on different links of cryodamage, providing a synergistic effect [11,17].

Plant-derived compounds of different chemical classes have previously been investigated as natural cryoprotectants for lactic acid bacteria. Wang et al. reported that soy polysaccharides increased the freeze-drying survival of *Lactiplantibacillus plantarum* AR113 by 19% compared with trehalose, whereas a combined system containing soy polysaccharides and trehalose provided a survival rate of 90.52% for *L. plantarum* WCFS1 and contributed to the preservation of membrane integrity and lactate dehydrogenase activity [35]. Plant-derived carbohydrates such as inulin and maltodextrin have also demonstrated protective effects during freeze-drying, although their ability to maintain bacterial viability during subsequent storage varied considerably [35]. More recently, oat-derived peptides were shown to mitigate freezing damage in *Lactobacillus delbrueckii* subsp. *bulgaricus* [36]. Compared with these plant-derived polysaccharides, carbohydrates, and peptides, basil extract represents a chemically distinct polyphenol-rich protective system. Its potential novelty is associated with the combination of antioxidant activity and membrane-associated interactions rather than protection based primarily on water replacement, glass formation, or regulation of ice formation. However, direct quantitative comparison between these studies is limited by differences in bacterial strains, preservation methods, storage conditions, and approaches used to assess viability.

The results of ROS detection additionally confirm the key role of oxidative stress in the cryoprotective activity observed. The 70% extract variant at a 1:30 dilution showed no increase in fluorescent signal compared with the control, consistent with its ability to minimize oxidative damage and maximize cell survival. The increased signal values in some variants may reflect both fundamental differences in ROS levels and the effects of phenolic compounds on the properties of the fluorescent probe, underscoring the need for careful interpretation of ROS detection in systems containing plant extracts [16,37].

Special attention should be paid to the dechlorophyllation stage of the extract, which involves freezing, followed by cold filtration. A significant decrease in optical density at 652 nm indicates effective removal of chlorophyll and associated lipophilic pigments. When the temperature decreases, solubility and aggregation of hydrophobic molecules occur, facilitating their precipitation and subsequent removal. This has not only technological but also functional significance, since chlorophyll is a well-known photosensitizer and, under certain conditions, can enhance ROS formation. Its removal can reduce the pro-oxidant contribution of the extract, thereby increasing the reproducibility and safety of the cryoprotective system [11,16].

In general, the results of this study allow us to consider basil extract a multifunctional natural cryoprotector, whose effect is based on a balance of antioxidant protection, membrane stabilization, and regulation of the physical state of the aqueous phase. Optimizing extract concentration and combining it with traditional cryoprotectors, such as sucrose, enables high cryoprotective efficiency while reducing potential toxicity, underscoring the promise of plant extracts for the development of bio-based cryoprotective systems for lactic acid bacteria.

## 4. Materials and Methods

### 4.1. Extraction

The selected ethanol concentrations and solid-to-solvent ratios were based on literature data on the extraction of phenolic compounds from basil and other plant materials [38,39]. Aqueous ethanol concentrations of 70–80% were chosen to provide effective extraction of phenolic acids and flavonoids with different polarities. Solid-to-solvent ratios ranging from 1:15 to 1:40 were examined to evaluate the influence of solvent volume on the total phenolic content, antioxidant activity, and co-extraction of chlorophyll, as well as to identify the most suitable extraction conditions. An ultrasonic bath at 20 °C for 15–20 min was used to extract polyphenolic substances. The samples were centrifuged at 4500 rpm for 30 min, and then the liquid phase was drained. The mixture was then filtered using filter paper, dechlorophyllated, and concentrated on a rotary evaporator at 45 °C and 30 mbar [40,41,42].

#### 4.1.1. The Method of Removing Chlorophyll from Extracts

The samples were frozen at −25 °C for 24 h, followed by cold filtration to remove coagulated chlorophyll. A spectrophotometer (Shimadzu UV-1800, Shimadzu Corporation, Kyoto, Japan) was used to measure the absorbance of the extract solutions at 652 nm before and after treatment.

Equation (1) was used to calculate the efficiency of chlorophyll removal:

(1) $$Chlorophyll\;removal\;efficiency\;\left(\%\right)=\;\frac{AbsB-AbsA}{AbsB}\times 100,$$

where *AbsB* is the absorption of the extract before the removal of chlorophyll, and *AbsA* is the absorption of the extract after the removal of chlorophyll [42].

#### 4.1.2. Determination of Antioxidant Activity by DPPH Method

The ability of ethanol basil extract to neutralize the DPPH radical was evaluated as previously described [43]. Samples of 1:50 extracts diluted with distilled water (200 µL each) were reacted with 2 mL of DPPH solution (0.1 mM), and absorbance was measured at 517 nm after 30 min of incubation in the dark at room temperature [12]. The percentage of DPPH inhibition was calculated using the following Formula (2):

$$DPPH\;inhibition\;\left(\%\right)=\;\frac{{Abs}_{contr}-{Abs}_{sample}}{{Abs}_{contr}}\times 100$$

The antioxidant content in the extract samples was determined from a linear graph between the percentage of inhibition and the concentration of ascorbic acid/Trolox: (y = 0.75955x − 2.33453, R^2^ = 0.9877)/(y = 339.35x + 12.08, R^2^ = 0.943).

#### 4.1.3. Determination of Phenolic Compounds by the Folin–Chocalteu Method

The amount of total phenols was determined using the Folin–Chocalteu reagent. A standard curve was constructed using gallic acid as a standard in the concentration range 10–120 mg/L, and the absorption was measured at 765 nm. Each extract sample was diluted 50 times with distilled water. A solution of 400 µL was mixed with 2 mL of 10% Folin–Chocalteu reagent, and then incubated at room temperature for 1 min. Next, 1500 µL of 7.5% sodium carbonate (Na_2_CO_3_) solution was added. The final mixture was shaken and incubated for 2 h in the dark at room temperature [44]. The phenol content in the extract samples was determined from a linear graph between the optical density (nm) and the concentration of gallic acid (y = 0.00898 + 0.01135x, R^2^ = 0.99952).

### 4.2. HPLC–ESI–MS/MS Analysis of Phenolic Compounds

Qualitative analysis of phenolic compounds in basil extracts was performed using high-performance liquid chromatography coupled with tandem mass spectrometry (HPLC–ESI–MS/MS). The analysis was carried out on a Shimadzu liquid chromatograph coupled with a triple quadrupole mass spectrometer LCMS-8060 (Shimadzu Corporation, Kyoto, Japan). Chromatographic separation was performed in reversed-phase mode using gradient elution at a flow rate of 0.320 mL/min; the total analysis time was 15 min. Detection was performed using an electrospray ionization (ESI) source. Phenolic acids (ferulic, caffeic, rosmarinic) and flavonoids (apigenin, luteolin) were analyzed in negative-ionization mode (ESI−), where deprotonated ions [M − H]^−^ were predominantly detected. Eugenol was analyzed in positive-ionization mode (ESI+), where protonated ions [M + H]^+^ were observed.

Ion detection was performed in multiple reaction monitoring (MRM) mode.

For verification of compound identification, reference standards of the corresponding compounds were used. Identification was qualitative and based on the agreement of retention times, MRM transitions, and mass spectral characteristics with those of standard compounds and literature data.

### 4.3. Quantum Chemical Modeling (DFT)

Quantum chemical calculations of the antioxidant potential of the studied phenolic compounds were performed in the ORCA 6.0.0 software package using the density functional (DFT) method. The molecular geometry was fully optimized using the hybrid B3LYP functional in combination with the def2-TZVP basis set [45]. The RIJCOSX exchange integral approximation scheme was used to speed up calculations, with strict convergence criteria in TightSCF and TightOpt. Dispersion interactions were accounted for using empirical Grimm’s D4 correction, which depends on the molecular charge [46].

The calculations were carried out both in the gas phase and with solvent effects included. The influence of the polar medium was modeled within the continuous dielectric CPCM framework, using parameters for ethanol [47].

After optimizing the geometries, harmonic-oscillation (Freq) calculations were performed to verify the stationarity of the optimized structures (absence of imaginary frequencies) and to obtain thermodynamic values. Based on the optimized structures, the energies of the highest occupied and lowest unoccupied molecular orbitals (HOMO and LUMO), the energy gap between them (HOMO–LUMO gap), which is an indicator of electronic stability, and the O–H bond-breaking energy (BDE) for phenolic hydroxyl groups were calculated. The latter parameter was considered a mechanism of hydrogen atom transfer (HAT) and a key thermodynamic descriptor of antioxidant activity [45].

To analyze the distribution of electron density and potential reaction centers, the electrostatic potential (MEP) on the isoelectronic surface was calculated. MEP maps were used to identify areas with increased nucleophilic or electrophilic activity and to locate those most likely to be involved in radical neutralization.

Input files were prepared, results were post-processed, and visualization was performed using the Multiwfn package (version 3.7) and IboView (http://www.iboview.org/), which are used to localize orbitals, analyze the electron density distribution, and build MEP maps. Three-dimensional visualization of structures, orbitals, and surfaces was performed in GaussView 6.0.

The three-dimensional structures of the studied compounds were obtained from the PubChem database in SDF format and then converted to a format suitable for quantum chemical calculations in ORCA.

### 4.4. Preparation of the Overnight Culture of Streptococcus thermophilus

An overnight culture of *Streptococcus thermophilus* strain NCDO 573 was obtained by inoculation of liquid medium M17 of the working culture of the strain. Cultivation was carried out at 37 °C for 15–18 h until the late exponential/early stationary growth phase was reached. Cellular biomass was deposited by centrifugation (Avanti J-30I, JA-18, Indianapolis, IN, USA) at 5000 rpm for 10 min (OD 600 1.8), and the filler fluid was removed. The resulting precipitate was used to prepare suspensions with cryoprotectors [48].

#### 4.4.1. Preparation of Cryoprotective Solutions

Saline solution (0.9% NaCl), 10% (m/v) sucrose solution, aqueous basil extracts pre-filtered through a membrane filter with a pore size of 0.22 µm and diluted with sterile distilled water in a ratio of 1:10, and mixtures of diluted basil extract with 10% sucrose solution prepared by mixing were used as cryoprotectors in a volume ratio of 1:1.

The precipitated biomass was resuspended, and then mixed with an appropriate solution at a biomass:cryoprotector ratio of 1:3. The resulting mixtures were frozen and stored at −25 °C for 1–7 days.

#### 4.4.2. Culture Seeding and Cell Viability

At the end of the freezing period, the samples were thawed at room temperature. For each cryoprotectant variant, tenfold successive dilutions were prepared in a sterile 0.9% NaCl solution. The first dilution was obtained by adding 100 µL of thawed sample to 9.9 mL of saline solution. Subsequent dilutions (up to 10^−6^) were prepared by transferring 1 mL of the previous dilution to 9 mL of 0.9% NaCl, pipetting carefully at each stage.

Deep seeding in dense nutrient media was used to determine the number of viable cells. From the selected dilutions, 100 µL of the corresponding bacterial suspension was added to sterile Petri dishes, followed by 15–20 mL of molten, cooled-to-45–50 °C, agarized M17 medium. The contents of the cups were thoroughly mixed in circular movements to distribute the cells throughout the thickness of the agar evenly, and the cups were left until the medium completely solidified. The cups were incubated at 37 °C for 48 h.

#### 4.4.3. Minimum Inhibitory Concentration of the Extract

The effect of basil extracts on the acid-forming activity of *Streptococcus thermophilus* was evaluated in a liquid M17 medium containing the pH indicator bromocresol purple (0.02 g/L), which changes from purple to yellow as the medium pH decreases. A standard overnight culture of *S. thermophilus* was grown in liquid medium M17 at 37–38 °C until the late exponential/early stationary phase was reached. The resulting culture was designated inoculum P2 and used for subsequent analysis. A series of working solutions in saline solution (0.9% NaCl) was prepared from the stock aqueous extract of basil with dilutions of 1:100, 1:75, 1:50, 1:25 and 1:1. For each extract concentration, 1700 µL of sterile liquid medium M17 with bromocresol purple and 100 µL of inoculum were added to the wells of the tablet of *S. thermophilus* (suspension P2), as well as 200 µL of the corresponding working solution of basil extract. A sample with an equivalent volume of saline solution added served as the control. The samples were incubated in a shaker incubator at 37–38.5 °C for 24 h, with the start time of the indicator’s color change fixed. The metabolic effect of the extract was assessed by the degree of suppression of the culture’s acid-forming activity relative to the control.

#### 4.4.4. Assessment of the Viability of *Streptococcus thermophilus* by Live/Dead Staining

The viability of *Streptococcus thermophilus* cells after freezing was assessed by fluorescent live/dead staining using LUCs 13 and YODI 3 dyes. After thawing the samples and preparing suspensions as described above, the cells were washed with phosphate-buffered saline (PBS) and resuspended in PBS (1970 µL). Working solutions of LUCs 13 (20 µL) and YODI 3 (10 µL) dyes were added to 10 µL of the cell suspension, thoroughly mixed, and incubated at 37 °C for 30 min in the dark. After incubation, the cells were washed with PBS to remove the external dye. Fluorescent images were obtained using a microscope with green and red fluorescence detection channels; cells stained with LUCs 13 were assessed as alive (green signal), and cells stained with YODI 3 were assessed as damaged/non-viable (red signal). At least 10 random visual fields were analyzed for each cryoprotector variant. The ImageJ program (version 1.54p; W. Rasband, National Institutes of Health, Bethesda, MD, USA), a scientific image processing and analysis software written in Java and available for various operating systems, was used to quantify the intensity of the fluorescent signal (https://imagej.net/ij/download.html (accessed on 31 December 2025)). Digital micrographs were imported into ImageJ without preprocessing. For each field of view, we split into color channels (Image → Color → Split Channels) and measured the average intensity (the Mean parameter) in the red and green channels over the entire image area using the standard Analyze → Measure function.

### 4.5. Molecular Dynamics Modeling of the Interaction of Low-Molecular-Weight Compounds with a POPC Membrane

Molecular dynamics (MD) simulations were performed using the GROMACS software package version 2025 [49]. The system under study was a bilayer membrane composed of 144 phosphatidylcholine (POPC) molecules, oriented in the XY plane and located at the center of a simulation cell measuring 7.0125 × 7.0125 × 10.0 nm^3^. The membrane was solvated with a TIP3P water model (~10,000 molecules) [50]. To achieve physiological ionic strength, Na^+^ and Cl^−^ ions were added to the system to a final concentration of 0.15 M. The molecules of the compounds under study (apigenin, ascorbic acid, eugenol, ferulic acid, glycerol, luteolin, rosmarinic acid, sucrose, Trolox) were parameterized using the CGenFF web service [51] and incorporated into the system by randomly replacing an equivalent number of water molecules. For each compound, the number of inserted molecules was chosen so that all systems contained approximately the same mass fraction of solute in the aqueous phase (~1 wt%). The systems were therefore matched by mass fraction rather than by equal molar concentration, and the resulting molar concentrations consequently differ between compounds (27.7–111.2 mM); the exact composition of every system is given in Table S1. Each compound was simulated in a separate system, and a compound-free POPC bilayer was simulated under identical conditions as a reference. The initial configuration of the system was prepared using the CHARMM-GUI service [52].

All simulations were performed using the CHARMM36 force field [53]. To account for electrostatic interactions, the PME algorithm was used with a direct space cutoff distance of 1.2 nm [54]. Van der Waals interactions were truncated at a distance of 1.2 nm using a force-switch modifier in a range from 1.0 to 1.2 nm. Bonds involving hydrogen atoms were limited by the LINCS algorithm [55].

The simulation procedure consisted of three main stages: energy minimization, equilibration, and production. Energy minimization was performed using the steepest descent method (5000 steps), with positional constraints applied to lipids and the molecules under study to eliminate steric clashes. The system equilibration procedure included two consecutive stages: temperature stabilization in the NVT ensemble (125 ps) and subsequent temperature and pressure stabilization in the NPT ensemble (1.125 ns). In both stages, a v-rescale thermostat was used to maintain a temperature of 303.15 K [56]. At the NPT stage, a C-rescale barostat was used to maintain a pressure of 1.0 bar in a semi-isotropic mode with an isotropic compressibility of 4.5·10^−5^ bar^−1^ [57]. Next, production modeling was performed in an NPT ensemble with a total duration of 20 ns and an integration step of 1 fs. All simulations were performed with relaxed positional constraints on the system components. The thermostatic and barostatic parameters at the production stage corresponded to those set at the equilibration stage. For each system, three independent replicas were simulated. The replicas differed in their initial atomic velocities, which were generated independently from a Maxwell–Boltzmann distribution at 303.15 K using a different random seed for each replica. Each replica underwent the full equilibration and production protocol described above, yielding a total production sampling of 60 ns per system.

The analysis of the obtained trajectories was performed using the MDAnalysis library for Python (https://www.python.org/) [58]. Data visualization was performed using the matplotlib and seaborn libraries.

To quantitatively assess the effect of the studied compounds on the properties of the lipid bilayer, the following parameters were calculated: membrane thickness (average distance between phosphorus atoms in opposite monolayers), area per lipid (average area in the XY plane per POPC molecule in the monolayer), spatial density profiles of various atomic groups, and orientation of lipid head groups (angle between the P-N vector and the membrane normal), as well as the number and stability of hydrogen bonds between compound molecules and lipid bilayer atoms.

### 4.6. Statistical Analysis

All experiments were carried out in three repetitions. The relative standard deviation of the detection results was calculated, and the experimental data were expressed as the average ± standard deviation. Statistical analysis was performed using software (Origin 2025, OriginLab Corporation, Northampton, MA, USA). Statistical significance was determined using univariate analysis of variance, and Duncan a posteriori tests. The differences were considered statistically significant at *p* < 0.05.

Molecular dynamics data were treated analogously. Each system was simulated in three independent replicas differing in their initial atomic velocities, which were generated from a Maxwell–Boltzmann distribution at 303.15 K using a different random seed for each replica. For each replica, the structural observables (membrane thickness, area per lipid, head-group tilt angle and number of hydrogen bonds) were averaged over the entire 20 ns production run. The three replica averages were treated as independent observations, and the results are reported as the mean ± standard deviation (SD, *n* = 3).

## 5. Limitations of the Study

Despite the encouraging results, the present study has several limitations that should be considered when interpreting the data and planning further work. The cryoprotective effect of basil extract was studied on a single model strain of lactic acid bacteria (*Streptococcus thermophilus*). In contrast, resistance to cryogenic stress and sensitivity to phenolic compounds may differ significantly between species and strains of microorganisms. Expanding research into other probiotic and technologically significant crops will allow us to assess the universality of the identified effects.

The extract was considered a multicomponent system, and the contribution of individual phenolic compounds to the cryoprotective effect was evaluated primarily indirectly, based on antioxidant activity and DFT modeling results. A direct experimental study of the effect of individual components and their combinations on cell survival could clarify the protection mechanisms and identify key active compounds.

Cryopreservation conditions were limited to one temperature regime (−25 °C) and a relatively short shelf life (up to 7 days). Lower temperatures and more extended storage periods are often used in industrial and collection practices, which require additional evaluation of the effectiveness of extracts across a broader range of cryogenic conditions. Oxidative stress and cell viability were assessed using fluorescent methods sensitive to the presence of phenolic compounds, which can affect probe properties. This imposes limitations on the quantitative interpretation of the data and underscores the need for additional independent methods of analysis in subsequent studies.

HPLC-ESI-MS/MS analysis was used only for the qualitative identification of phenolic compounds, while the total phenolic content of the extracts was quantitatively determined by the Folin–Ciocalteu method and ranged from 1350 to 2200 mg GAE/L. However, the concentrations of individual compounds were not determined. Therefore, the contribution of each identified phenolic compound to the observed antioxidant and cryoprotective effects cannot be quantitatively assessed. Further studies should include targeted quantitative HPLC-MS/MS analysis using reference standards and calibration curves for each compound, followed by direct evaluation of individual components and their combinations on bacterial cell survival.

## 6. Conclusions

It has been shown experimentally and theoretically that basil extracts (*Ocimum basilicum* L.) exhibit pronounced cryoprotective activity against *Streptococcus thermophilus*, depending on extraction conditions and concentration, due to their antioxidant and membrane-stabilizing properties. Extraction with water–ethanol systems (70–80%) yielded extracts with high phenolic content (≈1350–2200 mg GAE/L) and significant antioxidant activity, with the 80% extract showing the highest activity at a 1:15 ratio (5.6 mM Trolox). At the same time, a nonlinear relationship was observed between the total phenol content and radical-neutralizing ability, indicating the key role of the qualitative composition of the phenolic fraction. DFT modeling showed that luteolin and rosmarinic acid are characterized by minimal values of BDE O–H bonds in ethanol (~72–74 kcal/mol), comparable to or lower than those of ascorbic acid, which confirms their high antioxidant potential and is consistent with experimental data. The combined results suggest that the protective effect of basil extracts may arise from antioxidant activity together with membrane-associated interactions predicted by molecular dynamics simulations. Luteolin and rosmarinic acid were identified as promising contributors to this effect; however, their direct influence on bacterial membrane fluidity and organization requires further experimental confirmation. The use of basil extracts in cryoprotective media increased the survival rate of *S. thermophilus* after storage at −25 °C compared with saline solution and 10% sucrose. The most stable effect was observed with 70% extract at a 1:30 dilution and in the combined extract with sucrose systems, as confirmed by CFU counting and live/dead staining, reflecting better preservation of cell membrane integrity.

## Figures and Tables

**Table 1:** DFT-calculated parameters of the antioxidant potential of phenolic compounds: O–H bond-breaking energies (BDE, enthalpies, and free-energy values) in the gas phase and in ethanol, and HOMO–LUMO energy breaks for individual radical centers of molecules (luteolin, apigenin, ascorbic acid, eugenol, ferulic, and rosmarinic acids).

Compound	Radical	BDE (Enthalpy), kcal/mol	BDE Ethanol (Enthalpy), kcal/mol	BDE (Gibbs), kcal/mol	BDE Ethanol (Gibbs), kcal/mol	HOMO LUMO Energy Gap, E (Eh)	HOMO LUMO Energy Gap Ethanol, E (Eh)
Luteolin	OH_1_	71.01	73.47	71.56	73.93	0.151	0.147
	OH_2_	81.61	77.81	81.91	79.36		
	OH_3_	85.12	85.16	85.30	85.38		
	OH_4_	97.53	90.80	96.90	90.38		
Apigenin	OH_1_	97.64	90.85	96.99	90.48	0.153	0.151
	OH_2_	85.13	85.12	85.32	85.39		
	OH_3_	80.46	81.02	80.61	81.42		
Ascorbic acid	OH_1_	74.03	73.94	74.02	74.02	0.204	0.2
	OH_2_	75.58	73.56	76.00	76.00		
Eugenol	OH_1_	78.50	76.18	78.74	76.47	0.206	0.104
Ferulic acid	OH_1_	79.47	76.18	79.63	76.47	0.158	0.15
Rosmarinic acid	OH_1_	78.90	75.66	79.26	76.51	0.18	0.146
	OH_2_	70.64	73.55	71.90	74.44		
	OH_3_	81.29	75.86	81.90	76.44		
	OH_4_	72.00	71.51	72.86	72.35		

**Table 2:** Survival of *Streptococcus thermophilus* cells after freezing at −25 °C in various cryoprotective media with basil extract.

Sample Number	Conditions	CFU/mL	In Terms of ×10^7^	Log10 (CFU/mL)
1	70/30, 1/15 sucrose	7.1 × 10^7^	7.1	7.85
2	70/30, 1/15	3.7 × 10^7^	3.7	7.57
3	70/30, 1/30 sucrose	2.75 × 10^8^	27.5	8.44
4	70/30, 1/30	1.5 × 10^8^	15.0	8.18
5	80/20, 1/15 sucrose	6.7 × 10^7^	6.7	7.83
6	80/20, 1/15	2.9 × 10^7^	2.9	7.46
7	80/20, 1/30 sucrose	1.69 × 10^8^	16.9	8.23
8	80/20, 1/30	4.0 × 10^7^	4.0	7.60
9	NaCl	3.2 × 10^7^	3.2	7.51
10	Sucrose 10%	3.1 × 10^7^	3.1	7.49

## Data Availability

The original contributions presented in this study are included in the article. Further inquiries can be directed to the corresponding author.

## Supplementary Materials

The following supporting information can be downloaded at https://www.mdpi.com/article/10.3390/molecules31152661/s1. Table S1: Composition of the simulated systems: number of solute molecules added to the POPC/water box, and the resulting molar concentration and mass fraction in the aqueous phase.
