# Supplementary material for: Molecular Mechanisms of Basil (Ocimum basilicum L.) Polyphenol Extracts as Bio-Based Cryoprotectants for Streptococcus thermophilus: Chemical Profiling, DFT, Molecular Dynamics and Cell Viability
Source: Molecules. 2026 Jul 30;31(15):2661. doi: 10.3390/molecules31152661 (PMC13468213; doi:10.3390/molecules31152661)
Supplement: Supplementary file 1 [file molecules-31-02661-s001.zip › molecules-4407159-supplementary.pdf]

Supplementary materials

Molecular Mechanisms of Basil (*Ocimum basilicum* L.) Polyphenol Extracts as Bio-Based Cryoprotectants for *Streptococcus thermophilus*: Chemical Profiling, DFT, Molecular Dynamics and Cell Viability

Valeria A. Pyanchenkova [1], Vladislav S. Filozop [1], Mikhail O. Volodarskiy [1], Dmitrii N. Borovikov [1], Olga L. Balabanova [2], Olga O. Osmak [1], Semen S. Kazarin [1], Pavel V. Nesterov [1], Ivan V. Moskalenko [1], Mariia S. Ashikhmina [1,*] and Ekaterina V. Skorb [1]

[1] ITMO University, 9 Lomonosova St. 191002, St. Petersburg, Russia
[2] Saint-Petersburg Institute of emergency care named after I .I . Dzhanelidze, S. Petersburg, Russia
* Correspondence: msashikhmina@itmo.ru

Table S1. Composition of the simulated systems: number of solute molecules added to the POPC/water box, and the resulting molar concentration and mass fraction in the aqueous phase.

Molecule	Structural formula	N_{mol}	N_{water}	Molar concentration, mM	Mass ratio, wt%
Apigenin	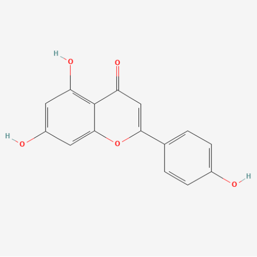 $C_{15}H_{10}O_5$	7	9999	38.9	1.04
Ascorbic acid	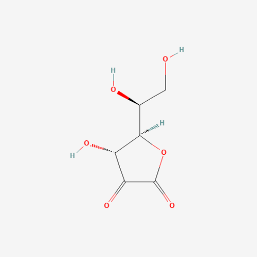 $C_6H_8O_6$	10	10010	55.4	0.97
Sucrose	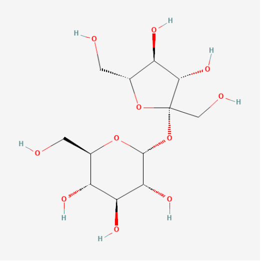 $C_{12}H_{22}O_{11}$	5	10019	27.7	0.94

Ferulic acid	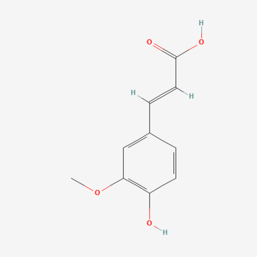 $C_{10}H_{10}O_4$	9	9998	50.0	0.96
Glycerol	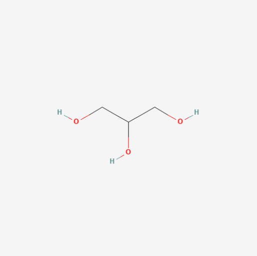 $C_3H_8O_3$	20	9987	111.1	1.01
Luteolin	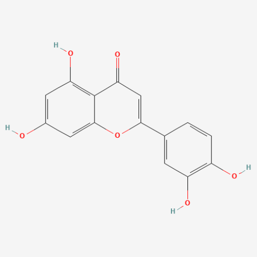 $C_{15}H_{10}O_6$	6	9960	33.4	0.95
Romaric acid	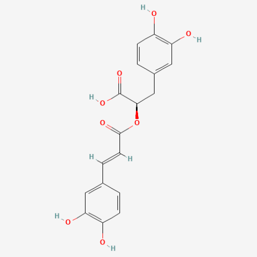 $C_{18}H_{16}O_8$	5	10003	27.7	0.99
Eugenol	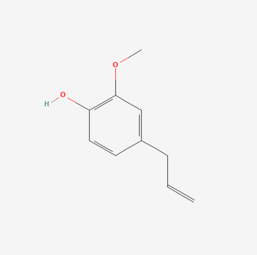 $C_{10}H_{12}O_2$	11	9978	61.2	0.99
Trolox	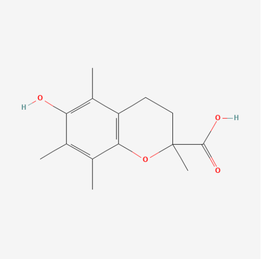 $C_{14}H_{18}O_4$	7	9999	38.9	0.96
